# Testable Hypotheses for Unbalanced Neuroimaging Data

**DOI:** 10.3389/fnins.2016.00270

**Published:** 2016-06-17

**Authors:** Martyn McFarquhar

**Affiliations:** Neuroscience and Psychiatry Unit, The University of ManchesterManchester, UK

**Keywords:** GLM, neuroimaging, unbalanced, ANOVA, SPM, FSL, sums of squares

## Abstract

Unbalanced group-level models are common in neuroimaging. Typically, data for these models come from factorial experiments. As such, analyses typically take the form of an analysis of variance (ANOVA) within the framework of the general linear model (GLM). Although ANOVA theory is well established for the balanced case, in unbalanced designs there are multiple ways of decomposing the sums-of-squares of the data. This leads to several methods of forming test statistics when the model contains multiple factors and interactions. Although the Type I–III sums of squares have a long history of debate in the statistical literature, there has seemingly been no consideration of this aspect of the GLM in neuroimaging. In this paper we present an exposition of these different forms of hypotheses for the neuroimaging researcher, discussing their derivation as estimable functions of ANOVA models, and discussing the relative merits of each. Finally, we demonstrate how the different hypothesis tests can be implemented using contrasts in analysis software, presenting examples in SPM and FSL.

## 1. Introduction

The general linear model (GLM) is a ubiquitous tool in neuroimaging, forming the basis of most common analysis approaches. Users of neuroimaging software packages are well placed to harness the power of the GLM given that tools such as FSL and SPM provide great flexibility in the forms of models that can be specified and the hypotheses that can be tested. Whilst largely advantageous, one drawback is that such flexibility demands the user understand in detail the underlying theory of the GLM. A part of this understanding is the concept of estimable functions in linear models, particularly in the case of unbalanced data. This is none more relevant than for the different forms of hypotheses that can be tested in unbalanced group-level ANOVA designs containing interactions. In the statistical literature there is a long history of debate around the relative merits of the Type I–III sums of squares. Despite this, there has seemingly been no discussion or clarity on the use of these different forms of hypothesis tests in neuroimaging. In addition, there exists no clear guidance on how such hypothesis tests could be implemented in popular software packages.

In this paper we present a guide for the neuroimaging researcher on the different forms of estimable functions that are possible in the case of unbalanced ANOVA models of neuroimaging data. We also present debate and opinion on the relative merits of each, emphasizing the hypotheses tested by each type in relation to the cell means of the design. To this end we begin with a review of the theory behind balanced ANOVA models in the GLM. This paves the way for the main discussion of unbalanced ANOVA designs, turning to the derivation of the different forms of sums of squares and their implementation using contrasts in neuroimaging software.

## 2. The balanced overparameterized ANOVA model in the GLM

The univariate GLM can be expressed as

$$\begin{array}{l} \text{Y}=\text{X}\theta +\epsilon \end{array}$$

where **Y** is the *n*×1 vector of observed values, **X** is the *n*×*k* design matrix, **θ** is the *k*×1 vector of parameters, and **ϵ** is the *n*×1 vector of errors. Solving for **θ** is usually achieved using the ordinary least-squares (OLS) estimator,

(1) $$\begin{array}{r} \hat{\theta }={\left({\text{X}}^{\prime }\text{X}\right)}^{-1}{\text{X}}^{\prime }\text{Y} \end{array}$$

assuming that **X**′**X** is invertible. These quantities are guaranteed the best linear unbiased estimates (BLUEs) under the conditions of the Gauss-Markov theorem (Christensen, 2011). Although this theorem does not require specification of a distribution for the data, such assumptions allow for the construction of hypothesis tests. It is therefore usual to assume that the data are drawn from a multivariate normal distribution, denoted **Y** ~ N(**Xθ**, σ^2^**I**), which is more usefully expressed in terms of the errors as

$$\begin{array}{l} \epsilon \sim N\left(0,\sigma^{2}\text{I}\right) \end{array}$$

Estimation of the single variance term proceeds from the model residuals

$$\begin{array}{l} {\hat{\sigma }}^{2}=\frac{1}{n-r}{\hat{\epsilon }}^{\prime }\hat{\epsilon } \end{array}$$

where *r* = rank(**X**) and

$$\begin{array}{l} \hat{\epsilon }=\text{Y}-\text{X}\hat{\theta } \end{array}$$

Taken together these results provide the basis for almost all the models typically used for neuroimaging data. Although this framework encompasses both subject-level and group-level neuroimaging models, here we focus solely on the group-level, specifically considering approaches such as the summary-statistic method for group-level modeling of neuroimaging data.

### 2.1. The 1-way ANOVA

In the 1-way case there is a single factor variable with *i* levels. Letting *Y*_*ij*_ indicate the measurement from the *i*th level for the *j*th subject (*i* = 1…*m, j* = 1…*n*_*i*_) the typical overparameterized 1-way model is

(2) $$\begin{array}{r} Y_{ij}=\mu +\alpha_{i}+\epsilon_{ij} \end{array}$$

where μ is the grand mean and α_*i*_ is the effect of group *i*. Here, *effect* denotes the difference between the grand mean and the mean of the group. As such, the cell mean for the *i*th group is given by

$$\begin{array}{l} \mu_{i}=\mu +\alpha_{i} \end{array}$$

This model is overparameterized because there are more parameters than cell means. In other words, if *i* = 1, 2, 3 then there are three unique model equations

$$\begin{array}{lll} \mu_{1} & = & \mu +\alpha_{1}\\ \mu_{2} & = & \mu +\alpha_{2}\\ \mu_{3} & = & \mu +\alpha_{3} \end{array}$$

with four unknowns (μ, α_1_, α_2_, α_3_). The consequence of this is that there are no unique solutions for the parameter values. As exemplified by Mumford et al. (2015), this is like trying to find 2 numbers that sum to 10. The choices are infinite.

Moving from the classical formulation of the ANOVA to its implementation within the GLM involves reconceptualizing the model in Equation (2) as a more generic regression model of the form

$$\begin{array}{l} Y_{ij}=\beta_{0}x_{0}+\beta_{1}x_{1}+\ldots \beta_{i}x_{i}+\epsilon_{ij} \end{array}$$

where β_0_ = μ and β_*i*_ = α_*i*_. The *x*_*i*_ predictors are typically indicator variables coding a 1 for group membership and a 0 otherwise. The *x*_0_ variable associated with the intercept is a 1 for all observations. An observation from group 1 would therefore render *x*_1_ = 1, with all other *x*_*i*_ set to 0. The model then simplifies to

$$\begin{array}{l} Y_{ij}=\beta_{0}+\beta_{1}+\epsilon_{ij}\\ \;=\mu +\alpha_{1}+\epsilon_{ij} \end{array}$$

returning us to the classical formulation of the ANOVA. In the GLM, the use of ANOVA models is therefore typified by a design matrix containing only indicator variables of ones and zeroes. If *i* = 1, 2, 3 the overparameterized 1-way ANOVA model can be expressed in the GLM as


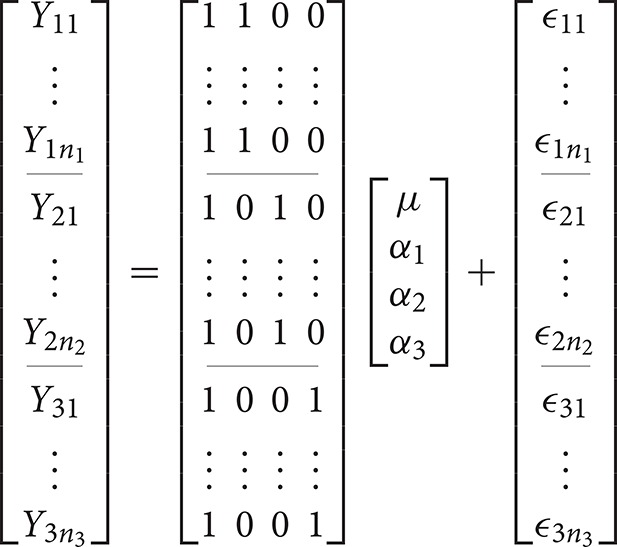


Here the inability to solve for the parameters can be demonstrated by considering that **X** is rank deficient so that (**X**′**X**)−1, from Equation (1), does not have a unique solution. This can be easily seen by considering that the constant is the sum of the other columns. As such, we have a situation of perfect multicollinearity.

### 2.2. The 2-way ANOVA

Although the 1-way ANOVA is the most basic and instructive case, it is only when considering models with interaction terms that many of the issues with unbalanced data become apparent. In the 2-way case there are two factors (denoted A and B). In a traditional crossover design the data are sampled from the intersection of the levels of the factors. The additive influences of the factors are termed the *main effects*, with the non-additive influence of the factors termed the *interactions*. These interaction effects can be expressed as γ_*ij*_ = μ_*ij*_ − (μ + α_*i*_ + β_*j*_), where μ_*ij*_ is the cell mean for level *i* of factor A and level *j* of factor B. Expressed in this form, it is clear that the interaction effect is simply the difference between the actual cell mean value and the expected cell mean value if the model were purely additive.

For subject *k* at the *i*th level of A and *j*th level of B the 2-way model equation is

$$\begin{array}{l} Y_{ijk}=\mu +\alpha_{i}+\beta_{j}+\gamma_{ij}+\epsilon_{ijk} \end{array}$$

where μ is the grand mean, α_*i*_ is the effect of the *i*th level of factor A, β_*j*_ is the effect of the *j*th level of factor B, γ_*ij*_ is the interaction effect, and ϵ_*ijk*_ is the error. As with the 1-way case, this model is overparameterized.

### 2.3. Estimable functions

As indicated earlier, the problem with overparameterized models is that there are no unique solutions for the parameter values. As an example, consider the 2-way model given in Equation (3).


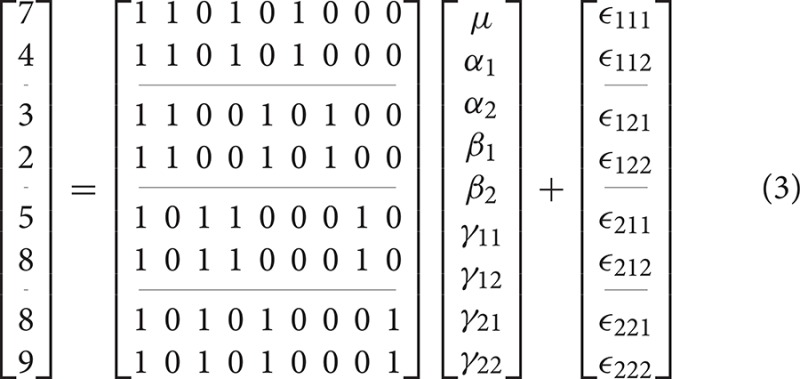


Four vectors of possible solutions for these parameters are given in Table 1. These solutions are, from left to right: solving Equation (1) using a pseudo-inverse of **X**′**X**, re-expressing the model using “treatment” coding, re-expressing the model using “sigma-restricted” coding and re-expressing the model using “cell-means” coding (see the Supplementary Materials). Although the existence of alternatives that render **X**′**X** invertible may suggest that the overparameterized formulation is overly complex and unnecessary, it is important to realize that non-overparameterized formulations (such as the coding schemes typically used for factors in explicit regression models) often lead to parameters that are more difficult to interpret. Such approaches are in fact equivalent to the overparameterized formulation with appropriate restrictions placed on the parameter values. As such we consider the overparameterized model as the most generic and didactically useful formulation of the ANOVA, despite its mathematical intractability.

**Table 1:** **Four possible vectors of solutions for the overparameterized 2-way ANOVA model**.

**Parameter**	**${\hat{\theta }}_{1}$**	**${\hat{\theta }}_{2}$**	**${\hat{\theta }}_{3}$**	**${\hat{\theta }}_{4}$**
μ	2.556	8.5	5.75	0
α_1_	0.111	−6	−1.75	0
α_2_	2.444	0	1.75	0
β_1_	1.444	−2	0.25	0
β_2_	1.111	0	−0.25	0
γ_1_	1.389	5	1.25	5.5
γ_2_	−2.778	0	−1.25	2.5
γ_3_	0.056	0	−1.25	6.5
γ_4_	2.389	0	1.25	8.5

Returning to the values in Table 1, it is notable that although they are all quite different they are all valid solutions as they all lead to the same fitted values

$$\begin{array}{l} X{\hat{\theta }}_{1}=X{\hat{\theta }}_{2}=X{\hat{\theta }}_{3}=X{\hat{\theta }}_{4}=\left[\begin{array}{c} 5.5\\ 5.5\\ 2.5\\ 2.5\\ 6.5\\ 6.5\\ 8.5\\ 8.5 \end{array}\right] \end{array}$$

Because there are multiple solutions that lead to the same estimated values, one may wonder what the worth is of any of the individual estimates given in Table 1? In truth, these values tell us nothing because they are dependent on the solution chosen. There are, however, certain linear combinations of the parameters that provide the same result irrespective of the solution. These linear combinations are known as *estimable functions*^1^.

An example of an estimable function for the 2-way model is,

(4) $$\begin{array}{r} \text{L}=\begin{array}{ccccccccc} {}[0 & 1 & -1 & 0 & 0 & 0.5 & 0.5 & -0.5 & -0.5] \end{array} \end{array}$$

as multiplying this vector by any one of the vectors of estimates in Table 1 produces the same result.

$$\begin{array}{l} \text{L}{\hat{\theta }}_{1}=\text{L}{\hat{\theta }}_{2}=\text{L}{\hat{\theta }}_{3}=\text{L}{\hat{\theta }}_{4}=-3.5 \end{array}$$

An example of a non-estimable function would be,

$$\begin{array}{l} L^{*}=\left[\begin{array}{ccccccccc} 0 & 1 & -1 & 0 & 0 & 0 & 0 & 0 & 0 \end{array}\right] \end{array}$$

as multiplying this vector by the different solutions in Table 1 produces different results.

$$\begin{array}{l} {\text{L}}^{*}{\hat{\theta }}_{1}=-2.3\\ {\text{L}}^{*}{\hat{\theta }}_{2}=-6\\ {\text{L}}^{*}{\hat{\theta }}_{3}=-3.5\\ {\text{L}}^{*}{\hat{\theta }}_{4}=0 \end{array}$$

As such, the result depends on the method of solving for **θ**. This is problematic, as all methods of finding solutions for the parameters rely on some form of constraint. As argued by Nelder (1994), such constraints should not be considered an intrinsic part of the model as our conclusions should not depend on the constraint. In the case of the GLM, only estimable functions can guarantee this.

More generally, any linear combination of the parameters coded in **L** is estimable if

$$\begin{array}{l} \text{L}=\text{T}\text{X} \end{array}$$

for some matrix **T** (McCulloch et al., 2008; Christensen, 2011). In words, this result indicates that any linear combination of the rows of **X** produces an estimable function due to the fact that the rows of **X** are estimable functions themselves. This is not surprising given that they dictate the predicted values of the model via *E*(**Y**) = **Xθ**. However, stating this explicitly leads to a particularly useful result, as any **L** is guaranteed estimable if it is constructed using a linear combination of the rows of **X**.

### 2.4. Some remarks on the different solutions to the ANOVA model

Throughout our examples, the scheme used to solve for parameters in overparameterized models is the pseudo-inverse of **X**′**X**. However, it should be recognized that alternative approaches exist. Indeed, the three other solutions given in Table 1 are derived from re-specifying **X** such that **X**′**X** is invertible. For neuroimaging, knowledge of these alternative approaches is particularly useful. In SPM it is usual to specify ANOVA designs using an **X** of full column rank, with overparameterized designs only occurring through the use of the Flexible Factorial module. In FSL, group-level models using FEAT do not allow a rank-deficient **X**. It is therefore necessary for the user to directly specify the structure of **X**, such that **X**′**X** can be inverted. Three common alternative approaches are discussed in the Supplementary Materials.

The use of different coding schemes provides a direct relationship between the presented ANOVA formulation and modern approaches that use an explicit regression formulation (such as linear and generalized linear mixed effects models). In these approaches, the coding of factors is done using a non-overparameterized scheme, and inference on the individual parameter estimates is often performed. A number of choices exist for the coding used, and each one has an impact on how the parameters, and the subsequent tests on the parameters, are interpreted. Here, a direct link with estimable functions can be made, as the tests on individual parameter estimates in a regression formulation is akin to the use of a single row of the identity matrix **I**_*k*_ as an **L**. For example, in a 2 × 2 treatment coded model, the contrast **L** = [ 0 1 0 0 ] cannot be formed from an estimable function of the overparameterized model. It is, however, equivalent to testing the single parameter coding factor A in an explicit regression using the same coding scheme. As the contrast is not estimable, care must be taken in interpreting the result of any test using it. Within an explicit regression model that contains a 2 × 2 factorial design, such a test on the single parameter for factor A does not actually produce the equivalent ANOVA main effect test, instead producing something more akin to a *simple* effect. As will be demonstrated later, one possible test to get the ANOVA main effect from a treatment coded design is **L** = [  0 1 0 0.5 ], which involves a linear combination of parameters, rather than a single test on an estimated value. Though this is clear from the ANOVA perspective, it is not always clear in explicit regression formulations that the coding used directly impacts the questions that are asked by the tests on the individual parameter estimates.

### 2.5. Hypothesis testing in ANOVA models

Although solving for parameters is one important aspect of ANOVA models, issues with unbalanced data are most readily seen in terms of performing hypothesis tests. Traditionally, the ANOVA hypothesis testing scheme is presented as a partitioning of the total sums of squares of the data into independent chunks. For a typical 2-way model, this partitioning can be expressed algebraically as

(5) $$\begin{array}{l} \sum_{i}\sum_{j}\sum_{k}{\left(Y_{ijk}-{\bar{Y}}_{\ldots }\right)}^{2}=\sum_{i}\sum_{j}\sum_{k}{\left({\bar{Y}}_{i..}-{\bar{Y}}_{\ldots }\right)}^{2}\\ \;+\sum_{i}\sum_{j}\sum_{k}{\left({\bar{Y}}_{.j.}-{\bar{Y}}_{\ldots }\right)}^{2}\\ \;+\sum_{i}\sum_{j}\sum_{k}({\bar{Y}}_{ij.}-{\bar{Y}}_{i..}-{\bar{Y}}_{.j.}\\ \;+\;{\bar{Y}}_{\ldots }{)}^{2}+\sum_{i}\sum_{j}\sum_{k}{\left(Y_{ijk}-{\bar{Y}}_{ij.}\right)}^{2} \end{array}$$

where Ȳ denotes a mean, and the dot notation indicates subscripts averaged over. The partitions on the right hand side of this expression are therefore those associated with factor A, factor B, the A × B interaction, and the error respectively (Searle, 1987). Dividing these quantities by their associated degrees of freedom produces *mean squares*, which are used to construct the ANOVA *F* tests.

The GLM hypothesis testing scheme used in neuroimaging software revolves around a slightly more general approach. Here, a sum-of-squares *Q* is constructed using,

$$\begin{array}{l} Q=\left(\text{L}\hat{\theta }\right)\prime {\left(\text{L}{\left(\text{X}\prime \text{X}\right)}^{-1}\text{L}\prime \right)}^{-1}\left(\text{L}\hat{\theta }\right) \end{array}$$

for a suitable hypothesis coded as an estimable function in **L**. No matter the coding used for **X**, the sums of squares in Equation (5) can all be constructed using suitable linear combinations of the estimated model parameters in **θ**. The only exception is the error sums of squares, which is constructed from the residuals. An *F* test can then be performed using

$$\begin{array}{l} F=\frac{Q}{r{\hat{\sigma }}^{2}} \end{array}$$

where *r* is the degrees of freedom for the hypothesis (the rank of **L**), and ${\hat{\sigma }}^{2}$ is the estimated residual variance of the model (the *mean square error*).

Within this more general testing framework, the hypothesis in question is coded in **L**. As such, understanding the question being put to the data involves interrogation the structure of **L**. As we will demonstrate, it is the form of **L** that dictates the different approaches used for unbalanced data. When using an overparameterized approach, however, it is often most useful to express the hypothesis in **L** in relation to the cell means, rather than in relation to the parameters. Taking an example of the **L** given in Equation (4), the hypothesis test given by **Lθ** can be written as

(6) $$\begin{array}{r} H_{0}:\alpha_{1}-\alpha_{2}+\frac{1}{2}\left(\gamma_{11}+\gamma_{12}\right)-\frac{1}{2}\left(\gamma_{21}+\gamma_{22}\right)=0 \end{array}$$

Replacing the interaction terms with their expanded form, the expression in Equation (6) can be simplified to

(7) $$\begin{array}{r} H_{0}:\frac{1}{2}\left(\mu_{11}+\mu_{12}\right)-\frac{1}{2}\left(\mu_{21}+\mu_{22}\right)=0 \end{array}$$

This hypothesis therefore equates to the average of the cell means for the first level of factor A, minus the average of the cell means for the second level of factor A. Although somewhat trivial for this example, this process will prove invaluable for understanding more complex estimable functions in unbalanced models later.

### 2.6. An alternative perspective on the ANOVA – model comparisons and *R*() notation

Before moving on to unbalanced designs, it is worth noting that there are in fact two equally useful conceptualizations of hypothesis tests in ANOVA models. Beyond understanding hypothesis testing in terms of the construction of a sum of squares *Q*, an alternative approach is via the concept of model comparison. Here the sums of squares for a hypothesis is seen as the difference in the sum of squared residuals for two competing models. In this approach, the sums of squares for a particular model term is seen as the reduction in error gained by the inclusion of that term in the model. Such an understanding then has an intuitive appeal, as the sums of squares can be seen as quantifying the degree of improvement in the model fit when additional terms are added.

A useful notation for indicating reductions in sums of squares due to model comparisons is the *R*() notation described by Searle (1987). Here a slightly simplified version of the *R*() notation is used in the interests of clarity. As an example, the reduction in the sum of squared errors found when fitting a model containing μ and α_*i*_ compared with a model containing only μ can be expressed as

$$R\left(\alpha_{i},\ldots ,\alpha_{k}|\mu \right)=R\left(\alpha |\mu \right)$$

Terms to the right of | are consistent in both models, and terms to the left of | are only contained in one of the models. Alternatively, this can be read as the effect of **α** after correcting for μ. When considering these tests in relation to the reduction in the sum of squared errors between two competing models, the *R*() notation can be similarly interpreted as

R(α|μ)=SSE(μ)-SSE(μ,α)

where SSE() denotes the sum of squared errors for a model containing the terms in brackets. An *F*-test can then be understood as taking the form

$$F=\frac{\text{SSE}\left(\mu \right)-\text{SSE}\left(\mu ,\alpha \right)}{r{\hat{\sigma }}^{2}}=\frac{R\left(\alpha |\mu \right)}{r{\hat{\sigma }}^{2}}$$

## 3. The unbalanced overparameterized ANOVA model

The theory behind ANOVA models is well described and understood for balanced data. Unfortunately, greater complexity is found when applying ANOVA models to data where the number of observations per cell differ. For these so-called unbalanced designs, much of the information provided from the balanced case remains relevant. The difference in the unbalanced case is that a clear decomposition of the total sums of squares into the constituent effects of the model is no longer possible. This is due to a loss of orthogonality between the ANOVA effects. This can be demonstrated by considering that the decomposition of sums of squares given in Equation (5) is only true in the balanced case (Searle, 1987). When the data are not balanced, the decompositions on the right hand side will not sum to the total on the left hand side. This indicates that the partitions do not constitute independent elements as the effects now overlap.

An example unbalanced dataset is given in Equation (8). A demonstration of the decomposition of the sums of squares for this dataset, and the preceding balanced dataset, is given in Table 2. These sums of squares were obtained using the decomposition in Equation (5). As it turns out, these sums of squares are those associated with tests for each effect as if it were the only effect in the model. For balanced data this is not problematic as the tests are orthogonal. For unbalanced data the lack of orthogonality means it is no longer sensible to consider an effect in isolation given that its sums of squares are no longer independent of the other terms. The only exception to this is the interaction term which, as will be demonstrated, never changes. It is therefore important to not blindly decompose the total sum of squares as would be done in the balanced case. Rather, the focus must be on sensible hypotheses, deriving the appropriate sum of squares and the corresponding estimable functions from there. Within the statistical literature there are 3 generally accepted approaches to deriving estimable functions in unbalanced ANOVA models, known as the Type I–III sums of squares. We note in passing that there is also a Type IV used for data with empty cells, however, given that no neuroimaging software accommodates empty cells we will not discuss them any further.


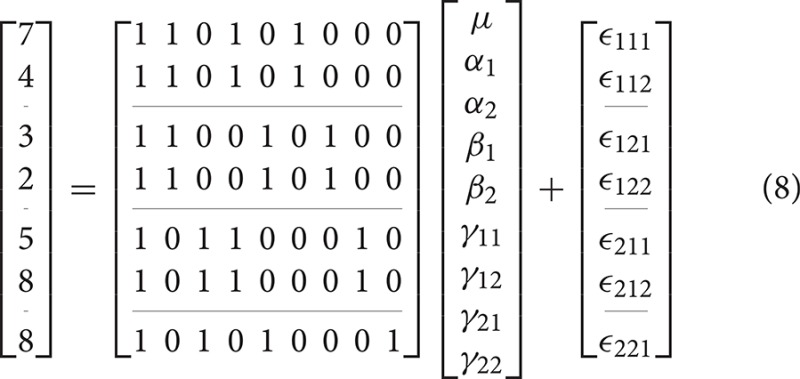


**Table 2:** **Example of the sums of squares derived from the traditional decomposition of the total into the constituent effects for the balanced and unbalanced data**.

**Sums of squares**	**Balanced**	**Unbalanced**
Total	47.5	35.43
A	24.5	15.43
B	0.5	4.76
A × B	12.5	8.6
Error	10	9.5
A + B + (A × B) + Error	47.5	38.29

Only in the balanced case does the total also equal the sum of the decomposed elements.

### 3.1. Type I sums of squares

Type I sums of squares, also known as sequential sums of squares, are those associated with the testing of each effect of the model in the order they are specified. In other words, this approach provides tests where each effect is only adjusted for those that precede it in the model equation. As such, the ordering of the model is important. This can be most readily understood using the *R*() notation detailed above, so that the Type I ANOVA table for the model *Y*_*ijk*_ = μ+α_*i*_ + β_*j*_ + γ_*ij*_ + ϵ_*ijk*_ is as detailed in Table 3.

**Table 3:** **The model comparisons that form the Type I sums of squares**.

**Effect**	**Sum of squares**
Constant	*R*(μ)
A	*R*(**α**|μ)
B	*R*(**β**|μ, **α**)
A × B	*R*(**γ**|μ, **α**, **β**)

For models with a natural ordering of terms, these tests can provide useful results given that each term added to the model is tested in relation to whether it provides greater predictive power than those terms that are already present. For regression models such tests are particularly useful, providing complimentary tests to those on the individual coefficients. For neuroimaging, these tests also provide the opportunity for a similar approach to stepwise regression modeling, without the necessity of fitting multiple models. However, it should be emphasized that better model building strategies exist, such as the use of Akaike Information Criterion (Akaike, 1998), and that the Type I tests simply provide a workable approach within the confines of the GLM testing procedures implemented in neuroimaging software.

In terms of the computation of the Type I tests, there are two approaches consistent with either the model comparison or estimable functions perspective. In relation to the interpretation of these tests, their sums of squares can be readily computed using the model comparisons detailed in Table 3. However, for neuroimaging applications, where both models must be fit at every voxel, this is a more demanding proposition. As such, it is also possible to specify these tests using an estimable function **L**. Though there are many ways to derive the weights of **L**, here we present a generic procedure based on an LU factorization of **X**′**X** using the Doolittle algorithm (Gaylor et al., 1970; Goodnight, 1979). See the Supplementary Materials for example MATLAB code implementing this approach for the purpose of calculating contrast weights. As an example, the coefficients for the unbalanced data presented above are given in Table 4.

**Table 4:** **Type I contrast weights derived from Doolittle factorization of X′X in the example unbalanced dataset**.

	**μ**	**α_1_**	**α_2_**	**β_1_**	**β_2_**	**γ_11_**	**γ_12_**	**γ_21_**	**γ_22_**
Constant	1	0.571	0.429	0.571	0.429	0.286	0.286	0.286	0.143
A	0	1	−1	−0.167	0.167	0.5	0.5	−0.667	−0.333
B	0	0	0	1	−1	0.6	−0.6	0.4	−0.4
A × B	0	0	0	0	0	1	−1	−1	1

Each row in Table 4 can be taken as an **L** matrix for testing the model effects in the order they appear in **X**. For example, the second row provides an **L** for testing the Type I main effect of factor A, where the effect of the constant has been removed. Similarly, the third row provides an **L** for testing the Type I main effect of factor B, where the effect of both factor A and the constant are removed. The final row contains the **L** for testing the interaction term. Notably this has not changed from its familiar form. If any effect spans more than 2 columns of **X** (such as factor with >2 levels) then there will be more than a single row containing weights for the effect. In these cases, **L** will be a matrix of weights consisting of the *k*−1 relevant rows from the Doolittle factorization (where *k* is the number of levels of the factor).

Although somewhat less intuitive than model comparison, the calculation of **L** allows a greater insight into the hypotheses being tested by the Type I approach. Consideration of only model comparison can lead one to conclude that such tests need only be considered on the basis of whether a comparison such as *R*(**β**|μ, **α**) is sensible in the context of the model. Although true in part, the weights used to calculate an expression such as *R*(**β**|μ, **α**) reveals one of the key disadvantages of this approach for unbalanced data, as the hypotheses are dependent on the cell frequencies. To see this, consider the **L** matrix for *R*(**α**|μ) presented in the second row of Table 4. Using the method detailed in Equations (6 and 7), this specification can be simplified to a more intuitive form, as shown in Equation (9).

(9) $$\begin{array}{r} H_{0}:\frac{1}{2}\mu_{11}+\frac{1}{2}\mu_{12}-\frac{2}{3}\mu_{21}-\frac{1}{3}\mu_{22}=0 \end{array}$$

Here it is clear that the weights associated with each cell mean depend on the cell frequency. In this case, each weight is derived from the number of subjects in the cell divided by the total number of subjects at that level of factor A. Intuitively this can be understood by considering that this **L** estimates a sum of squares equivalent to comparing two models that contains neither β_*j*_ nor γ_*ij*_. To get such a test from a model that *does* contain these terms, it is necessary to adjust the parameter estimates. As will be shown later, the procedure to do so is unavoidably dependent on the number of observations in each cell. The classical objection to this approach is therefore that the hypothesis can no longer be considered a testable statement about population parameters, unless the frequency in the sample is comparable to the frequency in the population (Searle, 1987). Furthermore, given its dependence on the order of the model terms, it is debatable how useful the hypothesis in Equation (9) actually is for a traditional ANOVA model. Although these insights appear damning of Type I tests, it is worth reiterating that this approach is legitimate and useful in cases of ordered regression models.

### 3.2. Type II sums of squares

Type II sums of squares are those associated with the testing of model terms under the assumption that higher-order effects containing those terms are zero. For example, when testing the main effect of A the A × B interaction is assumed zero. Unlike the Type I tests, however, the ordering of the model does not matter. As such the main effect of A is adjusted for B, and the main effect of B is adjusted for A. To make this clear, in a 3-way ANOVA model with effects A, B, and C the Type II main effect of A would be adjusted for B, C and B × C, but not A × B, A × C, or A × B × C as these effects contain A. This approach is shown from the model comparison perspective in Table 5.

**Table 5:** **The model comparisons that form the Type II sums of squares**.

**Effect**	**Sum of squares**
Constant	*R*(μ)
A	*R*(**α**|μ, **β**)
B	*R*(**β**|μ, **α**)
A × B	*R*(**γ**|μ, **α**, **β**)

Like the Type I sums of squares, the model comparison approach is relatively easy to implement via the model comparisons depicted in Table 5. From the estimable function perspective, Doolittle factorization of **X**′**X** can again be used, recognizing that this would need to be conducted multiple times with different **X**s. In each case, the effect of interest is added *after* terms for which it should be adjusted. If the model contains continuous covariates care must be taken to place the covariates before the factor of interest in **X** so that the sums of squares are also adjusted for the covariates. As such, there can be a large amount of model re-ordering in order to calculate the Type II weights correctly. A more generic procedure that does not require model re-ordering is detailed in the SAS algorithms (https://support.sas.com/documentation/). As an example, the Type II weights for the unbalanced data given earlier are shown in Table 6.

**Table 6:** **Type II contrast weights derived from Doolittle factorization of X′X in the example unbalanced dataset**.

	**μ**	**α_1_**	**α_2_**	**β_1_**	**β_2_**	**γ_11_**	**γ_12_**	**γ_21_**	**γ_22_**
Constant	1	0.571	0.429	0.571	0.429	0.286	0.286	0.286	0.143
A	0	1	−1	0	0	0.6	0.4	−0.6	−0.4
B	0	0	0	1	−1	0.6	−0.6	0.4	−0.4
A × B	0	0	0	0	0	1	−1	−1	1

In order to correctly calculate the weights for the main effect of factor A, the order of factor A and factor B must be swapped before performing the factorization of **X**′**X**.

Given the definition of the Type II tests, the only row that has changed in Table 6 compared to Table 4 is the row associated with factor A. The test coded in this row now produces a sum of squares that is adjusted for the intercept *and* factor B. As with before, the new hypothesis for factor A can be expressed in terms of the cells means, as shown in Equation (10).

(10) $$\begin{array}{r} H_{0}:\frac{3}{5}\mu_{11}+\frac{2}{5}\mu_{12}-\frac{3}{5}\mu_{21}-\frac{2}{5}\mu_{22}=0 \end{array}$$

In this form, one would be forgiven for thinking that the hypothesis does not look intuitive, let alone useful. However, as we will argue, framing a hypothesis test that reflects a model without interaction terms within a model that does contain these terms can be misleading. The hypothesis in Equation (10) is actually identical to

(11) $$\begin{array}{r} H_{0}:\alpha_{1}-\alpha_{2}=0 \end{array}$$

in the model *Y*_*ijk*_ = μ + α_*i*_ + β_*j*_ + ϵ_*ijk*_. From the model comparison perspective, we are therefore comparing,

(12) $$\begin{array}{rcl} Y_{ijk} & = & \mu +\beta_{j}+\epsilon_{ijk}\\ Y_{ijk} & = & \mu +\alpha_{i}+\beta_{j}+\epsilon_{ijk} \end{array}$$

as indicated in Table 5. Despite the seemingly unintuitive nature of this test when viewed from a cell means perspective, it is clear from both Equations (11 and 12) that such a test is readily understandable. As such, the slightly more involved arithmetic necessitated by the cell means model should not distract from the question that the test is posing. We shall return to this issue later.

#### 3.2.1. The principle of marginality

Though we have now covered the interpretation and calculation of the Type II tests, one may still wonder why such tests are of any interest at all. This is particularly as, given the weights in Table 6, similar caveats with the Type I approach exist as the tests appear dependent on the cell frequencies. There is, however, an appealing logic to these tests, known as the *principle of marginality* (Nelder, 1977, 1994; Nelder and Lane, 1995). In brief, this principle is based on the idea that interpreting a main effect in the presence of an interaction is uninformative. Indeed, some authors have gone as far as suggesting that any attempt to do so is “…an exercise in fatuity” (Kempthorne, 1952). Based on this logic, assuming that the interaction effect is zero is the only way to produce a meaningful test of a main effect. As such, main effects should only be adjusted for each other, as well as any interaction that does not contain the main effect in question. If it turns out that there is a significant interaction effect, the main effects should be ignored as a matter of course. As such, the fact that the model comparison in Equation (12) assumes no interaction is moot. As will be explained shortly, the Type III main effects tests can be interpreted in terms of adjustments of main effects for all other model terms. From the model comparison perspective, this involves testing models containing interactions *without* all their corresponding main effects. As such, the Type III tests implicitly entertain models that are arguably unrealistic. It is for this reason that the Type II tests are sometimes regarded more favorably than the Type III (Nelder, 1977, 1994; Nelder and Lane, 1995; Langsrud, 2003; Fox, 2008; Fox and Weisberg, 2011).

### 3.3. Type III sums of squares

Type III sums of squares are those associated with model comparisons in sigma-restricted models where only single terms are removed at a time. These are therefore tests where each effect is adjusted for *all* other model terms, thus violating the principle of marginality. Because of this, the model comparison perspective on the Type III tests is where much of the controversy surrounding the approach is found. However, the logic of the tests from the perspective of hypotheses about cell means makes the situation much clearer because these tests are the only ones that do not depend on the cell frequencies. These tests are also equivalent to Yates' weighted square-of-means approach (Yates, 1934), are often used by default in statistical software packages (e.g., SAS, SPSS, STATA), and correspond to the contrasts that researchers are taught to use within the GLM in neuroimaging.

The Type III tests are shown in Table 7 from the model comparison perspective. Here the notation from Searle (1987) is adopted to indicate two key points about these tests. Firstly, as stated above, the Type III tests based on model comparisons can only be considered for models using sigma-restricted coding (see the Supplementary Materials), a technicality discussed in Searle (1987). It is this seemingly arbitrary aspect of the Type III tests that has been used as an argument against their use (Venables, 1998), particularly given our earlier discussion of the fact that the model constraints should not influence the answers gained from the data. Here, both the Type I and Type II tests have an advantage, as their values do not depend on the constraint chosen for the model. Secondly, as indicated earlier, from the model comparison perspective the Type III tests of main effects involve comparing models with and without main effects, but maintaining all interaction terms. In comparison to the Type II approach, these tests do not treat the interaction effect as zero, rather they average over them. In the balanced case these two approaches are equivalent due to the orthogonality between the main effect and interaction tests. For unbalanced data this is not so. As an example, consider that the Type III main effect of factor A involves the following model comparison,

$$\begin{array}{lll} Y_{ijk} & = & \mu +{\dot{\beta }}_{j}+{\dot{\gamma }}_{ij}+\epsilon_{ijk}\\ Y_{ijk} & = & \mu +{\dot{\alpha }}_{i}+{\dot{\beta }}_{j}+{\dot{\gamma }}_{ij}+\epsilon_{ijk} \end{array}$$

and thus implicitly entertains a model that contains an interaction term with only one of the corresponding main effects. As argued by a number of authors, it is debatable how sensible this is (Venables, 1998; Langsrud, 2003; Fox, 2008; Fox and Weisberg, 2011).

**Table 7:** **The model comparisons that form the Type III sums of squares**.

**Effect**	**Sum of squares**
Constant	*R*(μ)
A	$R\left(\dot{\alpha }|\mu ,\dot{\beta },\dot{\gamma }\right)$
B	$R\left(\dot{\beta }|\mu ,\dot{\alpha },\dot{\gamma }\right)$
A × B	$R\left(\dot{\gamma }|\mu ,\dot{\alpha },\dot{\beta }\right)$

The dot notation indicates that the parameters adhere to sigma-restrictions.

As with all the other approach discussed so far, an **L** matrix can also be used the develop the Type III sums of squares. For the Type III tests this is particularly advantageous, as this approach is applicable to any coding of **X**. Again, the **L** weights can be derived using Doolittle factorization of **X**′**X**, but only after **X** has been reduced to its unique rows. This is therefore equivalent to calculation of these effects in the balanced case. Here we see the argument for using Type III tests emerging, as the **L** matrix used for Type III tests does not depend on the cell frequencies. Using this approach, the Type III weights for the example unbalanced dataset are shown in Table 8.

**Table 8:** **Type III contrast weights derived from Doolittle factorization of X′X in the example unbalanced dataset**.

	**μ**	**α_1_**	**α_2_**	**β_1_**	**β_2_**	**γ_11_**	**γ_12_**	**γ_21_**	**γ_22_**
Constant	1	0.5	0.5	0.5	0.5	0.25	0.25	0.25	0.25
A	0	1	−1	0	0	0.5	0.5	−0.5	−0.5
B	0	0	0	1	−1	0.5	−0.5	0.5	−0.5
A × B	0	0	0	0	0	1	−1	−1	1

Here the weights are derived after reduction of X to unique rows.

Looking at the main effect of factor A, the Type III test provides cell means comparison identical to Equation (6). As such, the hypotheses on the cell means do not differ between the balanced and unbalanced cases. As such the Type III tests can be interpreted as testing hypotheses that are generalizable, as they do not depend on the size of the sample. From this perspective, it has been argued that these tests are therefore the most appropriate for unbalanced data (Searle, 1987). However, this is not necessarily reason enough to dismiss the fact that this approach tests hypotheses that are arguably not sensible when considered from a model comparison perspective. Indeed, Nelder (1977) insists that the forms of models that the Type III tests actually compare are of “…no practical interest.” Such divergences in opinion make it clear why the difference between the Type II and Type III sum of squares remains such a contentious topic. This is particularly true given that the perspective one takes on such tests can lead to equally valid arguments for and against their use. As such, it is important to realize that none of the tests are “wrong” *per-se*, rather they are simply asking different questions.

### 3.4. Overview and merits of the type I–III tests

Now that the differences between the Type I–III tests has been covered, we turn to more general debate on their individual merits for hypothesis testing in classical ANOVA designs. Before doing so, we first present a concrete example to help elucidate the differences between how the means for the varying hypotheses are calculated in the Type I–III cases. Given that it is in the main effects tests that the various methods differ, it should be no surprise that it is the calculation of the marginal means that holds the key to understanding the different approaches.

#### 3.4.1. Calculation of marginal means in the type I–III tests

In Table 9 there are three data points from two hypothetical cells of data containing different numbers of observations. In row 1 we simply average all the data ignoring the cells, in row 2 we use equally weighted cell means, and in row 3 we use cell means weighted by the cell frequency. When the two cells are ignored, and the data are treated as coming from the same source, the results differ from when the cell means are averaged over. If instead the cell means are weighted by the cell frequency, the original row mean can be recovered. Herein lies the key conceptual differences between the Type III (equally weighted means) and the Type I–II (cell frequency weighted means) approaches. Here it is clear that the very process of correcting the cell means to recover the original row mean necessitates the use of the cell frequencies.

**Table 9:** **Demonstration of the differing approaches to producing marginal means in the presence of interaction effects using either equally weighted or frequency weighted cell means**.

**Approach**	**Cell 1**	**Cell 2**	**Result**
Averaged – ignoring cells	7	8,9	8
Equally weighted means (Type III)	$\frac{1}{2}\times 7$	$\frac{1}{2}\times 8.5$	7.75
Frequency weighted means (Type I–II)	$\frac{1}{3}\times 7$	$\frac{2}{3}\times 8.5$	8

#### 3.4.2. The type II vs. type III debate

As covered earlier, the Type I tests have limited utility beyond ordered regression models, where they should be considered as complimentary to the standard *t*-tests on the coefficient values. As such, much of the debate in the ANOVA literature relates to the choice between the Type II and Type III tests. Ultimately, much of this debate lies within ones feelings about the purpose of hypothesis testing in statistical models. As highlighted by Langsrud (2003) and Fox and Weisberg (2011), the ultimate aim of hypothesis testing should be the desire to answer specific and meaningful questions. Mathematically, this is expressed using linear combinations of the model parameters, however, inference on parameters without a foundation in meaningful questions is arguably counter to the purpose of statistical modeling. The reason that the Type II tests are often argued against is that they are considered solely in relation to parameters from models with interaction terms, rather than in consideration of the questions they ask. Indeed, the Type II tests of parameters from such models necessitate corrections in order to render their values equivalent to a model without interaction terms. This correction depends on the cell frequencies, but ultimately guarantees that the same question is being posed irrespective of the model form. As such, the question itself should therefore be the point of debate for the merit of the test, rather than the specific arithmetic details of how such a question can be posed across different models of the same data. From this perspective, the dependence on cell frequency in Equation (10) should not be considered an indictment about the worth of the hypothesis being tested, particularly as such a question is poorly framed in a model containing interaction terms. This ultimately highlights the limitations of only considering the ANOVA tests as hypotheses about the model parameters, particularly as this is arguably not the most important perspective on hypothesis testing. Such arguments weight heavily in favor of the the Type II tests over the Type III tests. In addition, the Type II tests of main effects are often more powerful than the Type III tests (Lewsey et al., 1997, 2001; Langsrud, 2003), a point of particular note for neuroimaging. As such, the Type II tests deserve serious consideration as the default approach for hypothesis testing in unbalanced ANOVA models. They are principled, sensible, and powerful tests that ask meaningful questions. Unlike the Type III tests, they do not depend on the model constraints when viewed from a model comparison perspective, and similarly, they do not implicitly entertain unrealistic models when testing main effects. When seen from the perspective of the questions posed to the data, it is difficult to argue against the approach taken by the Type II tests.

## 4. The unbalanced overparameterized ANOVA model in neuroimaging software

Now that the different approaches to dealing with unbalanced data in ANOVA models have been discussed, we turn to the practical application of these approaches in neuroimaging software. In this section we present the construction of the Type I–III tests in two of the most popular neuroimaging analysis software packages: SPM (www.fil.ion.ucl.ac.uk/spm/) and FSL (fsl.fmrib.ox.ac.uk/fsl/). Though we have limited examples to just these two packages, any analysis software that implements the mass-univariate GLM could be used. In all examples the model is a 2 × 2 between-subject ANOVA with cell counts as given in Table 10.

**Table 10:** **Group numbers for the example unbalanced neuroimaging data**.

		**A**	**A**	**Total B**
		**1**	**2**	
B	1	13	14	27
B	2	13	15	28
Total A		26	29	55

### 4.1. SPM

#### 4.1.1. Using the flexible factorial module

**Figure 1:**
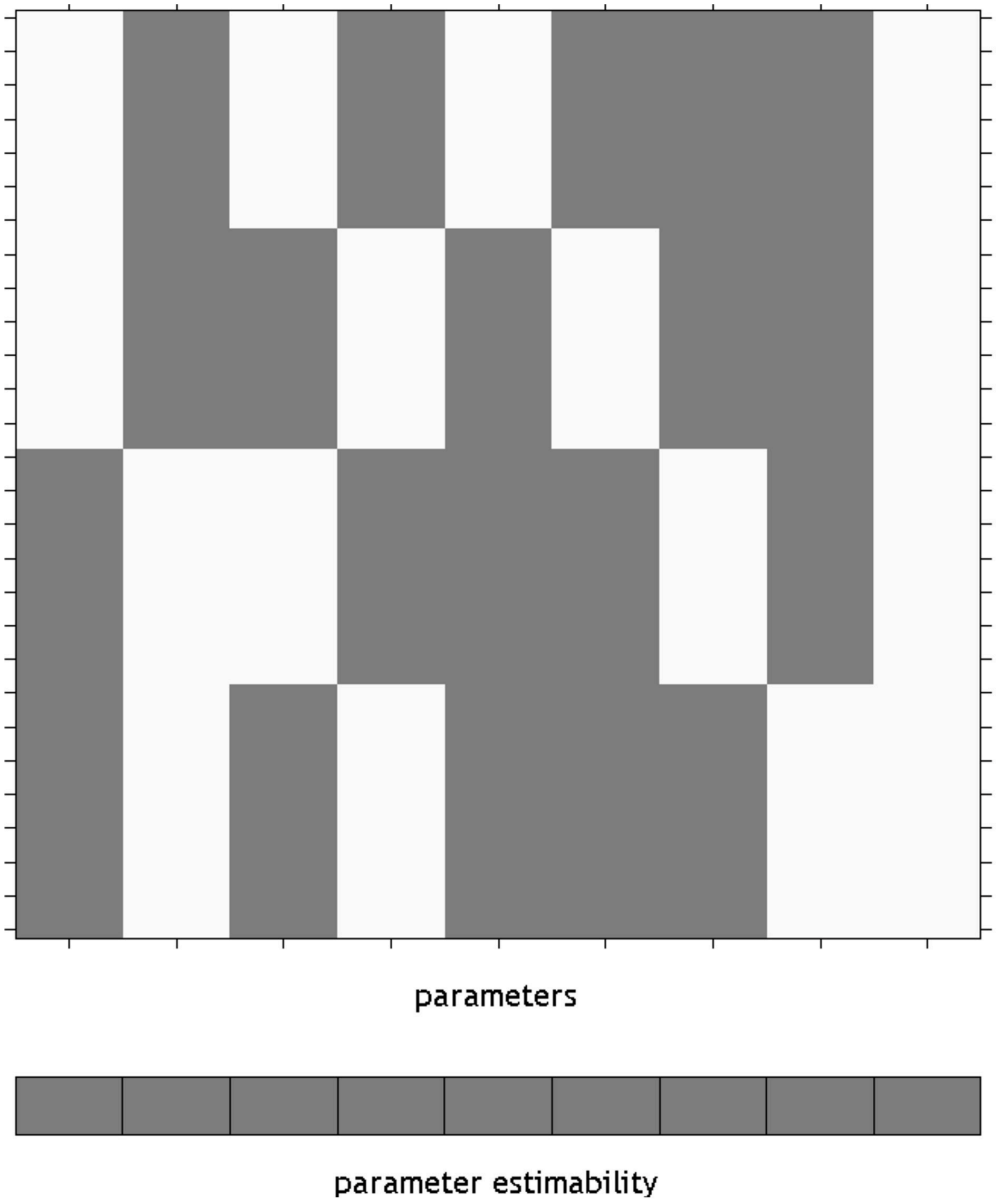
**The design matrix visualization for an overparameterized ANOVA model in SPM**. The use of an overparameterized ANOVA design is possible using the SPM Flexible Factorial module. Here, the blocks below the design matrix are gray, indicating that unique values for the parameters do not exist. The constant is placed on the end of the design as it is not included by default, rather it is added by specifying a covariate of ones.

Using an overparameterized design in SPM necessitates the use of the Flexible Factorial module. The SPM design matrix for the overparameterized ANOVA model is shown in Figure 1. Here a constant column has been added by specifying a covariate vector of ones. It is notable here that SPM has indicated that none of the parameters from this model will be unique, by providing a gray box per column below the design matrix. This is in keeping with the point made earlier about differing solutions in overparameterized designs, essentially highlighting that the values of the individual beta_^*^.nii images cannot be meaningfully interpreted. Once specified, this matrix is available in the SPM.mat file as X = SPM.xX.X, for the unfiltered design matrix. This is a convenience, as it is not necessary to specify the design matrix manually for use in a Doolittle factorization. It is, however, necessary to move the intercept column from last to first (e.g., X = [X(:,size(X,2)) X(:,1:size(X,2)-1)]).

**Figure 2:**
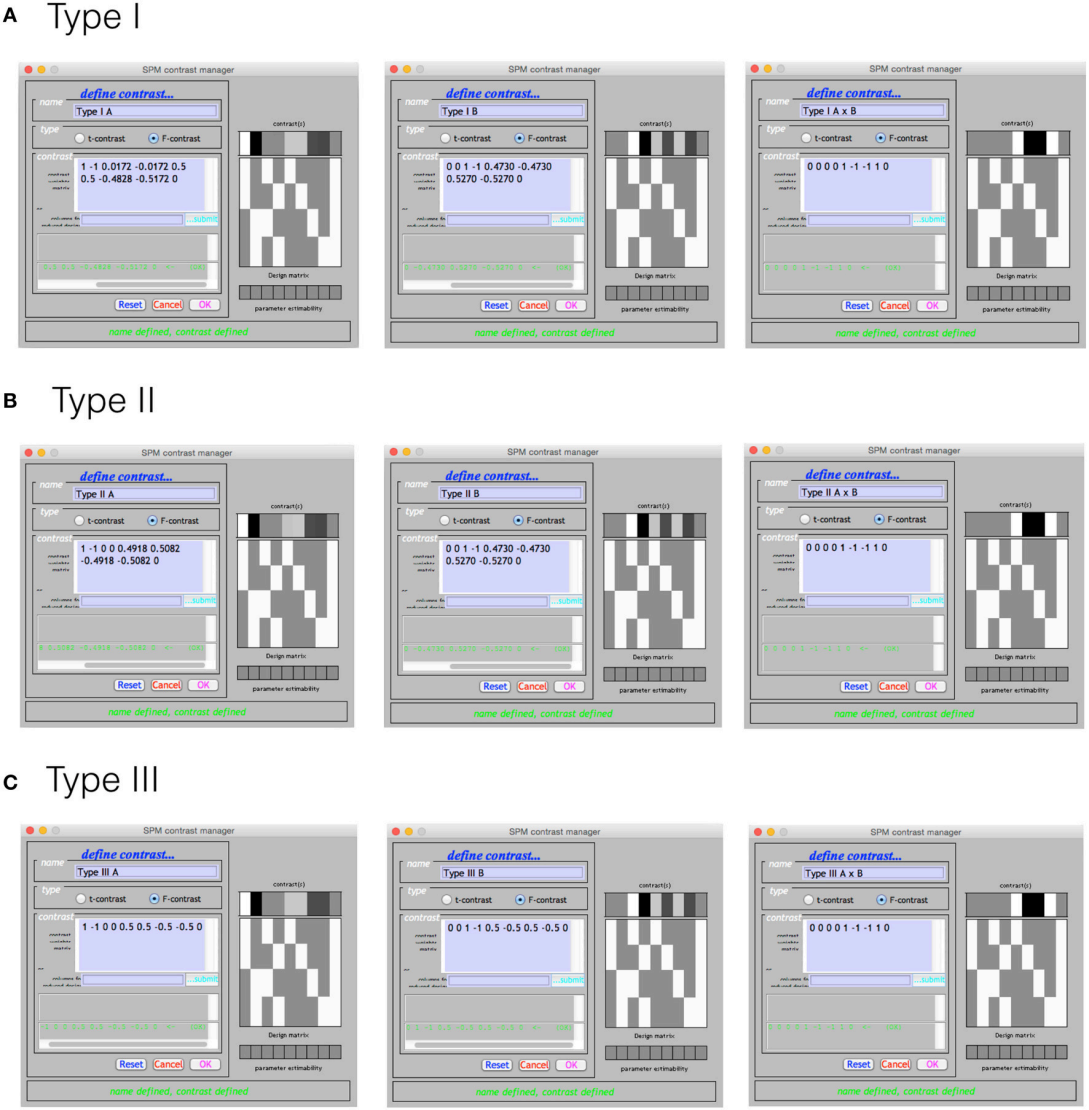
**The Type I–III hypotheses specified in the SPM contrast manager**. **(A)** Type I ANOVA weights **(B)** Type II ANOVA weights **(C)** Type III ANOVA weights. The green text below the input box for each example indicates that each contrast is an estimable function, as it has passed the estimability test used by SPM.

Using the Doolittle factorization of **X**′**X** provides scaled versions of the Type I weights for **L**. Using the MATLAB function given in the Supplementary Materials, this could be specified very simply as W = doolittleWeights(X). An example of specifying these effects in SPM is given in Figure 2A. Note that each of the contrasts were specified on a single line, but have been wrapped within the input box.

Derivation of the Type II tests follows much the same procedure. In this case the Doolittle factorization is performed twice, swapping the ordering of factor A and factor B in **X**. In this instance, the weights provided by the second decomposition are simply re-arranged so that they align with the original ordering of **X**. These weights are shown specified in the SPM contrast manager in Figure 2B. As expected, the **L** matrices for the interaction and main effect of B are identical to those in the Type I case.

As an additional point, it may be advantageous to make use of the *contrast masking* facility in SPM when exploring Type II main effects. Such an approach allows one to effectively “censor” voxels where a high-order effect is significant. As such, investigations of Type II main effects can be made only in voxels where the higher-order effects are suitably null. If there are multiple higher-order effects, a mask image of all significant higher-order effects could be used.

The Type III tests are those that will seem the most familiar to neuroimaging researchers. Their derivation from a Doolittle factorization is performed after reducing **X** to its unique rows. Again, using the MATLAB function given in the Supplementary Materials, this can be specified on a single line as W = doolittleWeights(unique(X, ‘rows’)). Because the Type III tests do not depend on cell frequency, they will be the same no matter the number of observations. These tests can therefore be constructed more generically using the number of cells rather than the frequencies within the cells. Generally speaking, this is much easier to do, and likely contributes to why the Type III contrast weights are generally taught for use in neuroimaging software. These tests are shown in the SPM contrast manager in Figure 2C.

#### 4.1.2. Using other SPM modules

**Figure 3:**
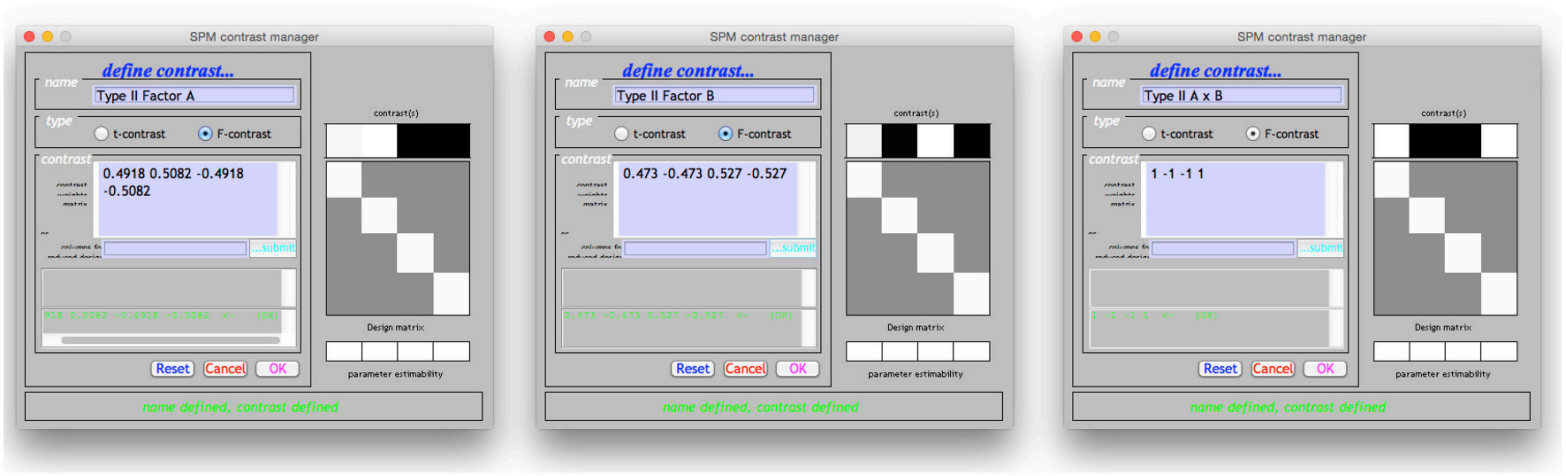
**Specification of the Type II tests for a cell means ANOVA in SPM**. The weights given in the contrast manager were derived from the overparameterized models given earlier. Although a more principled approach can be used to discern these weights, as shown in Equations (6 and 7), in this instance the weights were simply taken from the interaction terms in Figure 2B.

Although the above example made use of overparameterized designs in the SPM Flexible Factorial module, it is perfectly possible to derived the tests using any of the other models available in SPM. For example, if it is desirable to instead use the SPM Full Factorial module to specify a cell means design, then the contrasts already derived can easily be adjusted. The method used to convert an overparameterized contrast to a cell means contrast has already been demonstrated in Equations (6 and 7). However, for the current tests, it is more straightforward to simply take the weights associated with the interaction term and apply them to the cell means model. This is demonstrated for the Type II tests in Figure 3.

### 4.2. FSL

As mentioned earlier, FSL does not allow overparameterized designs to be used. This means that the overparameterized ANOVA model cannot be specified in FEAT directly. However, it is possible to start with an overparameterized model, calculate the estimable functions available to test the hypotheses of interest, and then convert these functions into ones useable in a model where **X**′**X** is invertible. Alternatively, both treatment and sigma-restricted design matrices can be submitted to Doolittle factorization to get the appropriate weights. The design matrix from an FSL FEAT group-level model is stored as a design.mat file in the corresponding ^*^.gfeat directory. This is a text file written in the FSL VEST format, and could be read into MATLAB using e.g., the palm_vestread() function from the Permutation Analysis of Linear Models (PALM) toolbox (http://fsl.fmrib.ox.ac.uk/fsl/fslwiki/PALM). The function in the Supplementary Materials could then be used to compute the Doolittle factorization of **X**′**X**, for example W = doolittleWeights(palm_vestread(‘./example. gfeat/design.mat’)). The appropriate weights from W can then be entered back into FEAT.

**Figure 4:**
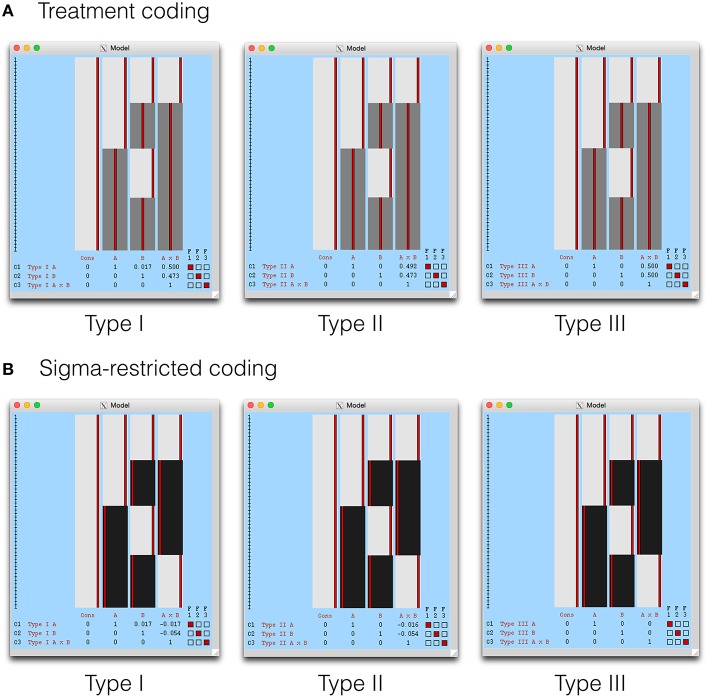
**The Type I–III hypotheses specified in FSL FEAT**. The models shown here include **(A)** treatment coding and **(B)** sigma restricted coding. These are both legitimate alternatives to the cell means model, used to render **X**′**X** invertible. The cell means model is not shown as it is identical to SPM, with weights calculated as indicated earlier.

The Type I-III tests are shown in the design visualization from FEAT in Figure 4. Given that the Type III tests from a sigma-restricted model are so straightforward, it is perhaps not surprising that this approach is recommended on the FSL GLM wiki (fsl.fmrib.ox.ac.uk/fsl/fslwiki/GLM). As the cell means model is identical to SPM, this has been omitted. In addition, *contrast masking* is also available in FEAT, and a such our comments on the Type II tests in SPM remain relevant for FSL also.

## 5. Summary

In this paper we have given an overview of the use of estimable functions in the GLM, paying particular attention to the different forms of hypothesis tests available for unbalanced ANOVA models. Though this issue has a long history in statistics, it has seemingly not been considered in the neuroimaging literature. Despite the fact that Type III tests have been settled on exclusively in popular software packages, there may be merit in the Type I and II tests for certain designs and approaches. Indeed, the Type II tests in particular provide greater power for investigating main effects, and arguably provide a more sensible hypothesis testing framework via the principle of marginality. Understanding the difference between these tests, and their derivation, allows for greater flexibility in the use of the GLM in neuroimaging, particularly in unbalanced designs. We have also touched on the use of Type I tests in regressions models, suggesting that these forms of hypotheses could be useful in ordered designs where the test of each coefficient is only adjusted for those that precede it. Using the overparameterized ANOVA model as a base, we have shown how all these tests can be derived from a generic framework that can be adjusted to suit any form of coding used. Such an approach allows immediate application irrespective of the software package used, but also provides a key focus on hypothesis testing as the single most important aspect of using the GLM with neuroimaging data.

## Author contributions

MM wrote the article, conceived the idea for the article, and produced the code used to create the figures and supplementary material for the article.

## Funding

This work was supported by a MRC Centenary Early Career Award (MR/J500410/1).

### Conflict of interest statement

The author declares that the research was conducted in the absence of any commercial or financial relationships that could be construed as a potential conflict of interest.

## Footnotes

^1^There is an element of subtlety here. The different parameter values given in Table 1 are not necessarily “meaningless,” rather their meaning changes depending on the solution chosen. As such, linear combinations that are not estimable can be thought of as posing a question that depends on the coding used in the design matrix. Their status as non-estimable simply refers to the fact that they cannot be universally applied to answer the same question. As such, these combinations are legitimate, so long as the parameter restrictions and coding are understood such that the question being posed is clear.
