# Supplementary material for: Testable Hypotheses for Unbalanced Neuroimaging Data
Source: Front Neurosci. 2016 Jun 17;10:270. doi: 10.3389/fnins.2016.00270 (PMC4911391; doi:10.3389/fnins.2016.00270)
Supplement: Supplementary file 1 [file DataSheet1.PDF]

Supplementary Material: Testable Hypotheses for Unbalanced Neuroimaging Data

Martyn McFarquhar*
*Correspondence:
Neuroscience & Psychiatry Unit, The University of Manchester, G.803 Stopford Building, Oxford Road, Manchester, M13 9PL, UK
martyn.mcfarquhar@manchester.ac.uk

1 ALTERNATIVE PARAMETERISATIONS OF ANOVA MODELS

1.1 Cell means coding

One approach to re-parameterising an overparameterised ANOVA model is through the use of "cell means" coding. To see the logic behind this approach, consider that the cell means in the overparameterised model are given by

$$\mu_{ij} = \mu + \alpha_i + \beta_j + \gamma_{ij}$$

so that it is possible to re-express the model such that it only contains a single parameter per cell mean using

$$Y_{ijk} = \mu_{ij} + \epsilon_{ijk}$$

Applying this in the context of the GLM leads to the following reduction of the design matrix and parameter vector from the example dataset given in **Equation 3** of the main text

$$\begin{bmatrix} 1 & 1 & 0 & 1 & 0 & 1 & 0 & 0 & 0 \\ 1 & 1 & 0 & 1 & 0 & 1 & 0 & 0 & 0 \\ 1 & 1 & 0 & 0 & 1 & 0 & 1 & 0 & 0 \\ 1 & 1 & 0 & 0 & 1 & 0 & 1 & 0 & 0 \\ 1 & 0 & 1 & 1 & 0 & 0 & 0 & 1 & 0 \\ 1 & 0 & 1 & 1 & 0 & 0 & 0 & 1 & 0 \\ 1 & 0 & 1 & 0 & 1 & 0 & 0 & 0 & 1 \\ 1 & 0 & 1 & 0 & 1 & 0 & 0 & 0 & 1 \end{bmatrix} \begin{bmatrix} \mu \\ \alpha_1 \\ \alpha_2 \\ \beta_1 \\ \beta_2 \\ \gamma_1 \\ \gamma_2 \\ \gamma_3 \\ \gamma_4 \end{bmatrix} \rightarrow \begin{bmatrix} 1 & 0 & 0 & 0 \\ 1 & 0 & 0 & 0 \\ 0 & 1 & 0 & 0 \\ 0 & 1 & 0 & 0 \\ 0 & 0 & 1 & 0 \\ 0 & 0 & 1 & 0 \\ 0 & 0 & 0 & 1 \\ 0 & 0 & 0 & 1 \end{bmatrix} \begin{bmatrix} \mu_{11} \\ \mu_{12} \\ \mu_{21} \\ \mu_{22} \end{bmatrix}$$

This is the approach typically used for ANOVA models in SPM, and is one choice available to FSL users.

1.2 Treatment coding

A second approach is the use of "treatment" coding. Here $\mathbf{X}$ is reduced to full column rank by first dropping the columns associated with one of the levels of each factor, and then re-forming the interaction columns by Hadamard (element-wise) multiplication. For a 2×2 example, the column associated with the second level of factor A can be dropped, as well as the column associated with the second level of factor B. This leave one column per-factor that can be used to form the new interaction column. In the GLM, this

leads to the following reduction of the design matrix and parameter vector from the example dataset given in **Equation 3** of the main text

$$\begin{bmatrix} 1 & 1 & 0 & 1 & 0 & 1 & 0 & 0 & 0 \\ 1 & 1 & 0 & 1 & 0 & 1 & 0 & 0 & 0 \\ 1 & 1 & 0 & 0 & 1 & 0 & 1 & 0 & 0 \\ 1 & 1 & 0 & 0 & 1 & 0 & 1 & 0 & 0 \\ 1 & 0 & 1 & 1 & 0 & 0 & 0 & 1 & 0 \\ 1 & 0 & 1 & 1 & 0 & 0 & 0 & 1 & 0 \\ 1 & 0 & 1 & 0 & 1 & 0 & 0 & 0 & 1 \\ 1 & 0 & 1 & 0 & 1 & 0 & 0 & 0 & 1 \end{bmatrix} \begin{bmatrix} \mu \\ \alpha_1 \\ \alpha_2 \\ \beta_1 \\ \beta_2 \\ \gamma_1 \\ \gamma_2 \\ \gamma_3 \\ \gamma_4 \end{bmatrix} \rightarrow \begin{bmatrix} 1 & 1 & 1 & 1 \\ 1 & 1 & 1 & 1 \\ 1 & 1 & 0 & 0 \\ 1 & 1 & 0 & 0 \\ 1 & 0 & 1 & 0 \\ 1 & 0 & 1 & 0 \\ 1 & 0 & 0 & 0 \\ 1 & 0 & 0 & 0 \end{bmatrix} \begin{bmatrix} \mu \\ \alpha \\ \beta \\ \gamma \end{bmatrix}$$

When using this coding it is important to recognise that the interpretation of the parameters changes. Most importantly, μ is now the mean of the cell formed from the levels of the factors that have been dropped. Each of the subsequent effects are then relative to this cell such that the parameters reflect the difference between the mean of the cell that has been dropped (often referred to as the "reference" or "base" group), and the mean of the grouping reflected in the columns of $\mathbf{X}$. Recovery of the cell means is as simple as looking at the coding of the rows in $\mathbf{X}$. For example, the cell given by level 1 of A and level 1 of B is $\mu + \alpha + \beta + \gamma$. Similarly, the cell for level 2 of A and level 1 of B is $\mu + \beta$. This is an important point, as it allows any contrast in this design to be constructed by starting with the definition of the cell means, and then constructing linear combinations of those cell means to form the hypothesis tests. Again, because this relates to linear combinations of the rows of $\mathbf{X}$, this approach is guaranteed to form estimable functions.

1.3 Sigma restricted coding

The final approach we will consider is the use of "sigma-restricted" coding, so-called because it employs the restriction that $\sum_i \alpha_i = \sum_j \beta_j = \sum_i \sum_j \gamma_{ij} = 0$. Here the approach is similar to the treatment coding as the columns associated with one level of each of the factors are dropped. However, rather than coding the dropped level as a 0, in sigma-restricted coding it is coded it as -1. Interaction columns are then formed using Hadamard multiplication. In the GLM, this leads to the following reduction of the design matrix and parameter vector from the example dataset given in **Equation 3** of the main text

$$\begin{bmatrix} 1 & 1 & 0 & 1 & 0 & 1 & 0 & 0 & 0 \\ 1 & 1 & 0 & 1 & 0 & 1 & 0 & 0 & 0 \\ 1 & 1 & 0 & 0 & 1 & 0 & 1 & 0 & 0 \\ 1 & 1 & 0 & 0 & 1 & 0 & 1 & 0 & 0 \\ 1 & 0 & 1 & 1 & 0 & 0 & 0 & 1 & 0 \\ 1 & 0 & 1 & 1 & 0 & 0 & 0 & 1 & 0 \\ 1 & 0 & 1 & 0 & 1 & 0 & 0 & 0 & 1 \\ 1 & 0 & 1 & 0 & 1 & 0 & 0 & 0 & 1 \end{bmatrix} \begin{bmatrix} \mu \\ \alpha_1 \\ \alpha_2 \\ \beta_1 \\ \beta_2 \\ \gamma_1 \\ \gamma_2 \\ \gamma_3 \\ \gamma_4 \end{bmatrix} \rightarrow \begin{bmatrix} 1 & 1 & 1 & 1 \\ 1 & 1 & 1 & 1 \\ 1 & 1 & -1 & -1 \\ 1 & 1 & -1 & -1 \\ 1 & -1 & 1 & -1 \\ 1 & -1 & 1 & -1 \\ 1 & -1 & -1 & 1 \\ 1 & -1 & -1 & 1 \end{bmatrix} \begin{bmatrix} \mu \\ \alpha \\ \beta \\ \gamma \end{bmatrix}$$

In this format, the interpretation of the parameters will again change. The parameter μ is now the *grand mean* of $\mathbf{Y}$. The remaining parameters therefore reflect differences relative to the grand mean, rather than differences relative to a reference cell of the design. As such, this approach is identical to the use of

deviation coding in an explicit regression model, as the individual tests on the parameter estimates are interpreted as deviations from the grand mean of the data. Similarly to the treatment coding, the cell means can also be recovered using the rows of $\mathbf{X}$ so that, for example, the cell mean for level 2 of A and level 1 of B is given by $\mu - \alpha + \beta - \gamma$.

2 MATLAB FUNCTION TO COMPUTE TYPE I WEIGHTS

Below we present an example MATLAB function to compute Type I weights from an overparameterised design matrix $\mathbf{X}$. This function implements LU factorisation of $\mathbf{X}'\mathbf{X}$ using the Doolittle algorithm. If $\mathbf{X}$ is full rank, the approach is identical to Doolittle factorisation, with the resultant upper-triangle matrix giving scaled versions of the Type I weights. If $\mathbf{X}$ is rank deficient, the algorithm skips rows with a 0 diagonal element. For Type II weights, the function must be used multiple times, re-ordering the effects in $\mathbf{X}$. For Type III weights from unbalanced designs, $\mathbf{X}$ must be reduced to only unique rows. This could be achieved using `W = doolittleWeights(unique(X, 'rows'))`. The function could also be used with treatment coded and sigma-restricted design matrices. For cell means design matrices, the weights should be derived from the overparameterised form of the design.

```
function U = doolittleWeights(X)
    A      = X'*X;
    tol    = 1e-15;
    [n, ~] = size(A);
    L      = zeros(n,n);
    U      = zeros(n,n);

    % Doolittle row elimination with redundant row skipping
    for k = 1:n
        L(k,k) = 1;
        for j = k:n
            U(k,j) = A(k,j);
            for s = 1:(k-1)
                U(k,j) = U(k,j) - L(k,s) * U(s,j);
            end
        end
        if U(k,k) > tol
            for i = (k+1):n
                L(i,k) = A(i,k);
                for s = 1:(k-1)
                    L(i,k) = L(i,k) - L(i,s)*U(s,k);
                end
                L(i,k) = L(i,k) / U(k,k);
            end
        end
    end

    % Remove 0 diagonal rows
    U(diag(U) < tol,:) = [];

    % Scale the weights
    for i = 1:size(U,1)
        U(i,:) = U(i,:) ./ max(U(i,:));
    end
end
```
